# The Integration of Cell Therapy and Biomaterials as Treatment Strategies for Remyelination

**DOI:** 10.3390/life12040474

**Published:** 2022-03-24

**Authors:** Eneritz López-Muguruza, Natalia Villar-Gómez, Jordi A. Matias-Guiu, Belen Selma-Calvo, Lidia Moreno-Jiménez, Francisco Sancho-Bielsa, Juan Lopez-Carbonero, María Soledad Benito-Martín, Silvia García-Flores, Natalia Bonel-García, Ola Mohamed-Fathy Kamal, Denise Ojeda-Hernández, Jorge Matías-Guiu, Ulises Gómez-Pinedo

**Affiliations:** 1 Laboratory of Neurobiology, Institute of Neurosciences, Hospital Clínico San Carlos Health Research Institute, Universidad Complutense de Madrid, 28040 Madrid, Spain; eneritzlom@gmail.com (E.L.-M.); nataliavillargomez@gmail.com (N.V.-G.); belselma@ucm.es (B.S.-C.); lidiamor-92@hotmail.com (L.M.-J.); j.lopezcarbonero@gmail.com (J.L.-C.); msbm65@gmail.com (M.S.B.-M.); sigarc12@ucm.es (S.G.-F.); natbonel@ucm.es (N.B.-G.); aolakamal@gmail.com (O.M.-F.K.); doddydenise@gmail.com (D.O.-H.); matiasguiu@gmail.com (J.M.-G.); 2 Department of Neurology, Institute of Neurosciences, Hospital Clínico San Carlos Health Research Institute, Universidad Complutense de Madrid, 28040 Madrid, Spain; jordimatiasguiu@hotmail.com; 3 Department of Physiology, Ciudad Real School of Medicine, Universidad de Castilla-La Mancha, 13005 Ciudad Real, Spain; francisco.sancho@uclm.es

**Keywords:** Multiple sclerosis, remyelination, cell therapy, biomaterials

## Abstract

Multiple sclerosis (MS) is a chronic degenerative autoimmune disease of the central nervous system that causes inflammation, demyelinating lesions, and axonal damage and is associated with a high rate of early-onset disability. Disease-modifying therapies are used to mitigate the inflammatory process in MS but do not promote regeneration or remyelination; cell therapy may play an important role in these processes, modulating inflammation and promoting the repopulation of oligodendrocytes, which are responsible for myelin repair. The development of genetic engineering has led to the emergence of stable, biocompatible biomaterials that may promote a favorable environment for exogenous cells. This review summarizes the available evidence about the effects of transplantation of different types of stem cells reported in studies with several animal models of MS and clinical trials in human patients. We also address the advantages of combining cell therapy with biomaterials.

## 1. Introduction

Multiple sclerosis (MS) is a chronic autoimmune disease of the central nervous system (CNS) that causes inflammation, demyelinating lesions, and axonal damage [1]. Treatment of the disease currently consists of the use of disease-modifying therapies (DMT) that modulate the inflammatory component of MS. These drugs reduce the number of relapses in patients with the relapsing-remitting form (RRMS); however, they are also associated with numerous adverse reactions, and most drugs do not prevent the axonal damage characteristic of progressive forms of the disease [2]. As a result, there is a need to develop drugs or therapies to promote remyelination.

Several studies report data suggesting that some DMTs contribute to remyelination; one example is fingolimod, a sphingosine-1-phosphate analog [3]. Although this drug promotes remyelination, it is also associated with adverse reactions [4]. Advances in the understanding of the factors and molecules regulating remyelination have given rise to the development of new treatments, which have been studied in several clinical trials. One of these is opicinumab, an antibody that antagonizes the LINGO-1 protein, a negative regulator of the process of remyelination [5]. Research is also being conducted into the role of molecules that positively contribute to myelin repair, such as vitamin D and thyroid hormones [6,7].

**Figure 1:**
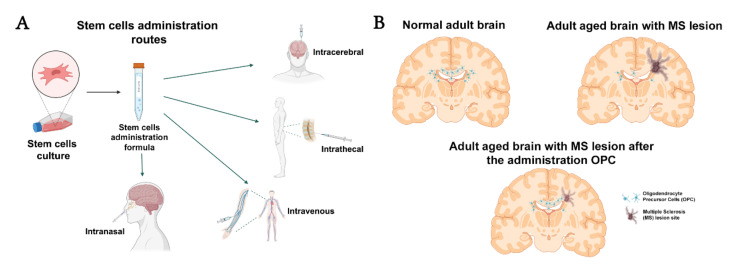
Graphical representation of the most employed administration routes for stem cell therapy (**A**), and oligodendrocyte precursor cells (OPC) distribution over the normal adult brain, adult aged brain where the OPC pool is reduced and presents an MS lesion, and adult aged brain with an MS lesion after the administration of OPC therapy (by any administration route), which enhances remyelination and, thus, the demyelinated zone is lessened (**B**).

The focus of treatment has shifted to target the promotion and stimulation of remyelination, a spontaneous regenerative process occurring after demyelinating lesions in diseases such as MS [8]. This process prevents axonal damage and results in clinical improvement. Nonetheless, its success depends on the contribution of several factors. After remyelination of these axons, areas called shadow plaques appear; these are characterized by reduced thickness of myelin sheaths, shorter internodal regions, and widened nodes of Ranvier [9]. For this phenomenon to occur, axons must not present signs of irreversible damage. Furthermore, there must be a sufficient population of oligodendrocyte precursor cells (OPC), which migrate to the lesion site, where they proliferate and differentiate into oligodendrocytes (Figure 1) [8].

Remyelination is more successful in RRMS than in progressive forms; there are several possible explanations for this. For instance, one of the factors that has received the most attention in the process of remyelination is age: magnetic resonance imaging (MRI) studies of patients with MS show decreased remyelination in older patients. Aging affects OPCs and their environment, contributing to a decline in remyelination. Most authors suggest that remyelination failure occurs in the differentiation of OPCs to oligodendrocytes since undifferentiated oligodendroglial-lineage cells have been identified in MS lesions [8]. However, several populations of mature oligodendrocytes have also been found in these lesions, indicating the possibility that specific populations may be responsible for remyelination and that the loss of these cells may contribute to remyelination failure [10].

Another factor closely related to remyelination is lesion type: MS presents with different types of lesions, where active lesions are characterized by the presence of mature oligodendrocytes, remyelination, and a larger population of phagocytic microglia; whereas inactive lesions present greater loss of oligodendrocytes and concentrated microglia at the edges of the lesion. Finally, mixed lesions present a greater reduction in remyelination [10].

The adaptive immune system plays an important role in the development of the disease, as one of the pathological characteristics of MS is the infiltration of autoreactive T cells across the blood-brain barrier (BBB). Inflammatory T cells, such as Th1 and Th17 cells, damage axons and myelin, whereas regulatory T cells promote remyelination [11].

In remyelination, other cell types play an essential role, such as:

### 1.1. Microglia

The microglia is necessary to generate a favorable environment for remyelination [12]. Firstly, these cells secrete growth factors that promote the proliferation and differentiation of OPCs (in the M2 polarization stage). They also phagocytize myelin debris from demyelinating lesions, which contain molecules that inhibit remyelination [13]. However, excessive levels of microglia presenting the inflammatory phenotype can lead to the overproduction of chemokines and reactive oxygen species, leading to oligodendrocyte death (M1 stage) [14].

### 1.2. Astrocytes

OPC activation and migration are mediated by astrocytes, which regulate the overexpression of fundamental transcription factors, including SOX2 and TCF4. In the context of a lesion, these cells are also capable of secreting fibroblast growth factor 2 (FGF2) and platelet-derived growth factor, which are involved in remyelination. However, astrocytes have also been shown to have a deleterious effect, as excessive activation leads to the formation of a glial scar, inhibiting the migration of oligodendrocytes to demyelinated areas [15,16].

### 1.3. Relationship between Microglia and Astrocytes

Astrocytes are thought to recruit phagocytic microglia to demyelinating lesions, as astrocyte-deficient mice have been shown to present higher levels of myelin debris, which may be explained by a reduction in the population of phagocytic microglia [15].

### 1.4. Circulating Factors

Several models of demyelination show problems with BBB permeability, and parabiosis studies have shown that remyelination may be influenced by soluble factors. The factors involved in remyelination in the context of a demyelinating lesion include FGF21, apelin, and cholesterol. Fibrinogen has also been associated with inflammation and axonal damage [11].

In recent years, cell therapy has emerged as a significant advance in the treatment of MS, with several animal studies showing promising results. The combination of different types of cells has been shown to be efficacious both for reducing inflammation and for cell replacement in demyelinated areas and in the process of remyelination. However, proper integration of these cells in the body depends on the cellular microenvironment; therefore, cell therapy has progressed in line with the development of biomaterials.

This review addresses the different types of cells that have been explored in the treatment of demyelinating lesions and diseases, such as MS. The selection of the bibliography was carried out using the PRISMA reporting guidelines methodology (Supplementary Material S1).

## 2. Cell Therapy and Types of Cells

### 2.1. Hematopoietic Stem Cells

Immunoablation followed by autologous hematopoietic stem cell transplantation (I/AHSCT) is a procedure that has been extensively studied in the treatment of autoimmune diseases, including MS. This practice consists in mobilizing hematopoietic stem cells (HSC) from the bone marrow to the peripheral blood, followed by immunoablation and subsequent repopulation of the immune system by infusion of autologous HSCs. This therapy is appropriate for patients with RRMS who present active inflammation and poor response to DMTs. The method fundamentally targets the inflammatory component of MS, as it is associated with a decrease in the concentration of autoreactive T cells and a reduction in the number of relapses and MRI lesions. However, I/AHSCT is associated with immunosuppression-related adverse reactions (Table 1), which have led to the death of the patient in some cases [17]. Given its therapeutic effects in patients with RRMS, new protocols are being developed to guarantee total safety for patients.

One disadvantage of this technique is the fact that it exclusively modulates the inflammatory component of MS; therefore, other cell types must be used to promote regeneration.

### 2.2. Non-Hematopoietic Stem Cells

Non-hematopoietic stem cells (nHSC) are a class of progenitor stem cells derived from the bone marrow that can differentiate into non-hematopoietic cell lineages. The first non-hematopoietic mesenchymal stem cells (MSCs) were discovered by Friedenstein in 1976, who described clonal, plastic adherent cells from bone marrow capable of differentiating into osteoblasts, adipocytes, and chondrocytes. More recently, investigators have now demonstrated that multipotent MSCs can be recovered from a variety of other adult tissues and differentiated into numerous tissue lineages, including myoblasts, hepatocytes, and reprogramming them to neural line tissue. As a result, the therapeutic use of MSC is now the most widely used nHSCs in preclinical and clinical trials. Several methods are currently available for isolation of the MSCs [18,19,20].

#### 2.2.1. Mesenchymal Stem Cells

MSCs are the most widely used nHSCs in clinical and preclinical trials due to their pluripotency and capacity to differentiate into cells of non-mesodermal lineage (Table 1). Part of the success of MSCs is due to the ease of obtaining them from allogeneic tissues, increasing their availability, and avoiding immune rejection by the patient [21]. Studies suggest that in patients with MS, implanted MSCs present unique properties that assist in suppressing inflammation, inhibiting oxidative stress, and in neuroprotection, neuroregeneration, and remyelination.

Their anti-inflammatory effect is associated with their capacity to inhibit the activity of autoreactive T cells and stimulate the release of anti-inflammatory cytokines [22]. Furthermore, in a study using the experimental autoimmune encephalitis (EAE) animal model, Witherick et al. [23] demonstrated the role of MSCs in modulating cytotoxic reactive oxygen species through the secretion of the extracellular antioxidant molecule superoxide dismutase 3. This molecule eliminates excess superoxide produced by the microglia after demyelination, improving symptoms of the disease.

Another beneficial effect reported with MSC administration is the secretion of such neurotrophic factors as nerve growth factor, ciliary neurotrophic factor, and brain-derived neurotrophic factor (BDNF). In an animal model of EAE, administration of MSCs is reported to increase levels of these molecules, leading to neurological improvement [24]. Furthermore, MSCs have been shown to secrete paracrine factors via extracellular vesicles containing cytokines, chemokines, and growth factors. Given their capacity to cross the BBB, they may be used as vehicles for therapeutic factors. In a model using Theiler’s murine encephalomyelitis virus, administration of extracellular vesicles derived from MSCs demonstrated the high capacity of these vesicles to migrate to lesion sites. This led to an increase in levels of white matter-associated markers, such as myelin basic protein and 2’,3’-cyclic-nucleotide 3’-phosphodiesterase [25].

Despite the benefits of using these cells, they have also been associated with an increased risk of cancer [26].

#### 2.2.2. Adipose-Derived Stem Cells

Positive results have been reported for the administration of adipose-derived stem cells (ASC) in the EAE model, both at early stages and in the chronic phase. Several studies agree that neuromodulation is the main action mechanism of these cells, although their administration has also been associated with increased levels of OPCs in demyelinated areas [27]. All studies with the EAE animal model report that the injection of these cells reduced disease severity and improved motor function. Other reported effects include decreases in demyelination and inflammatory infiltration in the damaged areas [28]. Most intravenously administered ASCs cells accumulate in the lymphoid organs, with fewer cells migrating to the CNS; thus, they modulate the inflammatory response through resident and peripheral macrophages. In fact, treatment with these cells has been shown to increase levels of cytokines associated with macrophages with anti-inflammatory phenotypes, such as IL-10, and to reduce levels of those associated with autoreactive T cells [29].

Despite the benefits demonstrated in the mouse EAE model, no clinical trial has been conducted to confirm these effects in patients with MS. However, the administration in human trials has shown that the implanted cells cause few adverse effects [30]. Therefore, further studies are needed to demonstrate the potential of these cells as a treatment for MS.

#### 2.2.3. Neural Stem Cells

Neural stem cells (NSC) have been studied extensively in the treatment of various diseases of the CNS. These cells are obtained from embryonic stem cells (ESCs); in adults, they persist in the subgranular zone of the hippocampus and in the subventricular zone of the lateral ventricles [31]. NSCs can differentiate into neurons, astrocytes, and oligodendrocytes; however, their success depends on the ability to secrete neurotrophic factors and to modulate the immune response [32]. In the EAE model, administration of NSCs is associated with increased levels of OPCs in demyelinated areas, which has been correlated with increased remyelination [33]. Transplantation of these cells also increases the number of regulatory T cells, which are needed to eliminate or mitigate the presence of autoreactive T cells [34]. NSCs also release such neurotrophic factors as leukemia inhibitory factor, associated with regeneration [35]. In vitro studies have also found these cells to activate the microglia and to promote the expression of molecules associated with the anti-inflammatory M2 microglial phenotype over the proinflammatory M1 phenotype [36].

It is still not possible to use these cells in the clinical context; clinical trials are being conducted in patients with MS, although the results have not yet been published (Table 1).

#### 2.2.4. Oligodendrocyte Precursor Cells

OPCs have been considered for cell therapy due to their capacity to differentiate into oligodendrocytes and to secrete neurotrophic factors. The availability of these cells is limited, and they present ethical problems due to the fact that they are mainly obtained from ESCs [37]. As an alternative, they may be obtained from the umbilical cord or from undifferentiated pluripotent cells (e.g., induced pluripotent stem cells (iPSC)) [38,39]. Human ESC-derived OPCs secrete such neurotrophic factors as transforming growth factor-beta 2 and BDNF, which may regulate the survival of OPCs and promote regeneration [40,41]. OPCs have also shown promising results in animal models of MS, including EAE and encephalomyelitis induced with the JHM strain of the mouse hepatitis virus. In both cases, transplantation of OPCs reduced the clinical severity of the disease and decreased demyelination [39,42]. In the EAE model, transplantation also considerably increased remyelination [39]. Despite these promising findings, OPCs also present disadvantages, such as difficulty reaching the lesion site [37].

A recent study with human oligodendroglioma cells showed that the intranasal route is viable for administering cells, paving the way for a promising new scenario for cell therapies for MS [43,44].

To date, no clinical trial has studied the use of these cells to treat MS, although one trial used them to treat patients with cervical spinal lesions. However, all patients presented adverse reactions; thus, additional clinical trials are needed before these cells can be used in the clinical context (Table 1).

#### 2.2.5. Oligodendrocytes

Oligodendrocytes have been used in cell therapy due to their potent myelinating effect. However, few studies have been conducted in models of MS [45,46]. Oligodendrocytes derived from iPSCs are known to present the same transcriptomal and phenotypic characteristics as mature oligodendrocytes. In a study by García-León et al. [47], oligodendrocytes were derived from iPSCs obtained from fibroblasts. Increased myelin production was observed after implantation of oligodendrocytes into shiverer mice. Furthermore, oligodendrocytes have been obtained from iPSCs in patients with MS with a view to their implantation and use in cell therapy in the near future. Similar results were reported in another study in which implantation of oligodendrocytes resulted in high survival, proliferation, and differentiation to mature oligodendrocytes [48].

#### 2.2.6. Induced Pluripotent Stem Cells

While MSCs and NSCs have received the greatest attention in the clinical context of MS, iPSCs also show great therapeutic potential. In a study using the EAE animal model, iPSCs were obtained from mouse fibroblasts and subsequently differentiated to neural progenitor cells (NPC) in vitro. Transplantation of these cells into mice with EAE showed that the NPCs were able to differentiate into neurons, which were able to integrate into the damaged tissue. This, in turn, resulted in the mitigation of disease symptoms and improved the animals’ mobility due to a reduction in the infiltration of T cells, which probably decreased the recruitment of cytotoxic lymphocytes [49].

In addition to its therapeutic effect, iPSC technology has enabled the generation of functional astrocytes presenting the CD49f marker. Astrocytes positive for this marker were associated with neuronal growth and cytokine secretion in the context of inflammation. When they were stimulated with TNF-α and IL-1β, they acquired a reactive phenotype mimicking one of the characteristics of these cells in MS [50]. This finding may serve as a model for the development of new therapies.

This technology has also been applied in human samples. In one study, peripheral blood mononuclear cells were extracted from healthy controls and patients with RRMS and primary progressive multiple sclerosis (PPMS). Cells were used to obtain iPSCs and subsequently differentiated to NPCs. The iPSCs derived from patients presented reduced PAX6 expression, associated with difficulty differentiating to subsequent lineages. These cells also showed more marked cellular senescence, a process involved in pathological cellular stress and inflammation [51]. Given the involvement of these two processes in demyelination in MS, it is essential to seek to better understand these results, as they may lead the way to new therapies based on the modulation of cellular senescence.

However, iPSCs have been demonstrated to be potentially tumorigenic, as they have been linked with the generation of the oncogene *c-Myc*. Furthermore, in diseases such as MS, in which the cell environment is hostile, we need to guarantee that the grafted pluripotent cells are able to differentiate and regenerate in that microenvironment [52].

## 3. Biomaterials and Cell Therapy. Generation of Biohybrids

Cell therapy for the treatment of CNS diseases often fails due to the suboptimal cellular microenvironment. To address this problem, these cells are administered in combination with biomaterials intended to replicate the physiological conditions needed for the proliferation, migration, differentiation, and survival of the implanted cells. These biomaterials can be generated from different sources and usually mimic the characteristics of the extracellular matrix.

### 3.1. Hydrogels

**Figure 2:**
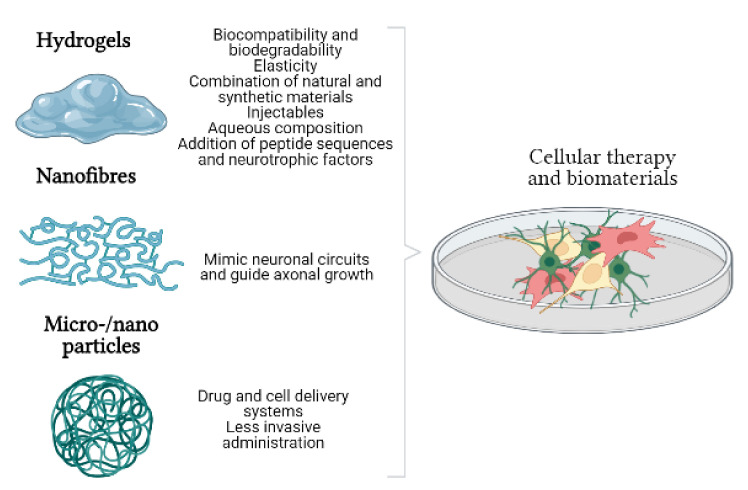
Types of biomaterials used in cell therapy and their properties in the context of central nervous system repair.

Hydrogels constitute one of the most widely used alternatives for the repair of CNS lesions due to their biocompatibility, biodegradability, and high flexibility (Figure 2). The main component of these biomaterials is water, and they can be formed with natural and synthetic materials. Natural materials include hyaluronic acid (HA), fibrin, chitosan, and alginate, whereas the most widely used synthetic materials include polyethylene glycol (PEG) and other polymers as polylactic acid (PLA) and polyglycolic acid. One advantage of using natural components is the prevention of rejection reactions, although they are combined with synthetic materials as it is easier with the latter to add peptide domains and bioactive molecules. However, hydrogels are also highly malleable, and their mechanical properties may be adapted to the environment in which they will be implanted [53].

The use of hydrogels in mouse and rat models of spinal cord injury has demonstrated how biomaterials can improve the efficiency of cell therapy with such cells as MSCs, NSCs, and OPCs. In vitro studies have shown the capacity of numerous materials to promote cell survival and differentiation, as well as myelination. However, few in vivo studies have been conducted. One example is the study conducted by Li et al. [54], who developed a hydrogel composed of HA and PEG-crosslinked human recombinant gelatin containing OPCs. Administration of the hydrogel in an ethidium bromide-induced demyelination model was associated with greater OPC survival and greater remyelination of spinal cord axons.

### 3.2. Nanofibres

The CNS comprises a highly complex circuit of cells, with axons being the most specific. The orientation of axons is essential to guiding neuronal growth and promoting regeneration (Figure 2). In the context of MS, Hoveizi et al. [55] designed a scaffold composed of PLA and chitosan as a treatment for EAE. The scaffold contained PC12 cells, which were differentiated to neural-like cells and implanted into the lateral ventricles of the brain. The treatment was associated with a clinical improvement; electron microscopy showed that demyelinated axons were fewer in number and presented better morphology in the treated animals.

### 3.3. Micro- and Nanoparticles

Another disadvantage of cell therapy in the repair of CNS lesions is the difficulty of reaching the lesion site. Due to the potential invasiveness of administering cells by direct injection to the lesion site, micro- and nanoparticles are under development to enable delivery by other routes. Research into these particles has mainly focused on the release of drugs or other molecules in models of MS (Figure 2).

In the cuprizone-induced demyelination model, for example, administration of immobilized chondroitinase ABC I on porous silicon nanoparticles reduced the demyelinated area and astrogliosis in the corpus callosum [56].

In another model of MS using lysolecithin-induced demyelination, intraperitoneal injection of curcumin-loaded nanoparticles was found to reduce inflammation and demyelination [57]. However, fewer studies have used these particles to transport cells, with most using in vitro models. The combination of cell therapy with micro- and nanoparticles seems to be a highly promising strategy whose continuing development may lead to clinical applications in the near future.

## 4. Conclusions

The great efforts made to develop treatments for MS have produced a broad spectrum of therapies that alleviate symptoms and reduce the relapse rate. However, the immunomodulatory mechanisms of these drugs cause adverse reactions, and they do not promote regeneration. Therefore, exploiting cell therapy in the treatment of MS represents a novel perspective. Despite how new this field is, results from animal models of MS have demonstrated the therapeutic potential of these cells. However, most treatments are currently in the experimental phase, and more clinical trials are needed to verify the effect of these cells in human patients. There can be no doubt that cell therapy holds great promise in the very near future, whether through modulating inflammation to promote a favorable microenvironment for remyelination or through the use of OPCs to repopulate endogenous niches or replace endogenous cells whose populations may be reduced due to genetic or age-related factors. This will enable remyelination to be restored. In pathological conditions, the implanted cells are not correctly integrated, possibly due to inflammation or increased levels of reactive oxygen species. For this reason, advances in tissue engineering have enabled the development of biomaterials that may improve the outcomes of cell transplantation, whether in the form of hydrogels, scaffolds, or encapsulation of cells and/or growth factors to favor their survival. Furthermore, these materials may be administered by safer, less traumatic routes, such as the intranasal route.

In conclusion, we are close to achieving a cell therapy or biohybrid (biomaterial/cells) for the treatment of such demyelinating diseases as MS. There is a clear need for development in preclinical phases to guarantee its safety and enable its translation to clinical contexts, where these strategies may enable personalized treatment, with each patient receiving treatment and a biomaterial aiming to promote remyelination.

## Figures and Tables

**Table 1:** List of clinical trials for each cell type.

Cell Type	Disease	Clinical Trial Code	Patients	Procedure	Date	Results
**HSCs**	MS	NCT00273364	*n* = 110 18–55 years	Immunosuppression followed by autologous hematopoietic cell transplantation	Completed 5 January 2017–30 August 2019	Severe adverse reactions: febrile neutropenia, hypophosphatemia, hypokalemia, hyperglycemia
**MSCs**	MS	NCT04823000	*n* = 24 18–65 years MS (RRMS, SPMS, PPMS)	Multiple intrathecal or intravenous transplants of autologous MSCs at 6–12 month intervals	Completed 1 January 2013–1 April 2020	No published results
	MS	NCT01377870	*n* = 22 18–55 years RRMS	Intravenous transplantation of MSCs	Completed June 2011–April 2014	No published results
**NSCs**	MS	NCT03269071	*n* = 4 18–55 years Progressive MS	Intrathecal transplantation of 4 different volumes of NSCs	Completed 17 May 2021–31 July 2021	No published results
	MS	NCT03282760	*n* = 24 18–60 years SPMS	Intraventricular transplantation of different volumes of NSCs for 3 months followed by immunosuppression for 6 months	Completed 9 September 2017–29 May 2021	No published results
**OPCs**	Cervical spinal cord injury	NCT02302157	*n* = 25 18–69 years	AST-OPC transplantation. A total of 5 cohorts (3 dosages: one injection of 2 million or 10 million cells; or 2 injections of 10 million cells)	Completed March 2015–December 2018	Adverse reactions: urinary tract infection, sepsis

HSC: hematopoietic stem cells; MS: multiple sclerosis; MSC: mesenchymal stem cells; NSC: neural stem cells; OPC: oligodendrocyte precursor cells; PPMS: primary progressive MS; RRMS: relapsing-remitting multiple sclerosis; SPMS: secondary progressive multiple sclerosis.

## Supplementary Materials

The following supporting information can be downloaded at: https://www.mdpi.com/article/10.3390/life12040474/s1, Figure S1: chart PRISMA.
