# Supplementary material for: The Integration of Cell Therapy and Biomaterials as Treatment Strategies for Remyelination
Source: Life (Basel). 2022 Mar 24;12(4):474. doi: 10.3390/life12040474 (PMC9027199; doi:10.3390/life12040474)
Supplement: Supplementary file 1 [file life-12-00474-s001.zip › life-1647005-supplementary.pdf]

life

MDPI

Review

The Integration of Cell Therapy and Biomaterials as Treatment Strategies for Remyelination

Eneritz López-Muguruza [1,†], Natalia Villar-Gómez [1,†], Jordi A. Matias-Guiu [2], Belen Selma-Calvo [1], Lidia Moreno-Jiménez [1], Francisco Sancho-Bielsa [3], Juan Lopez-Carbonero [1], María Soledad Benito-Martín [1], Silvia García-Flores [1], Natalia Bonel-García [1],Ola Mohamed-Fathy Kamal O [1], Denise Ojeda-Hernández [1], Jorge Matías-Guiu [1,2] and Ulises Gómez-Pinedo [1,*]

Supplementary Materials:

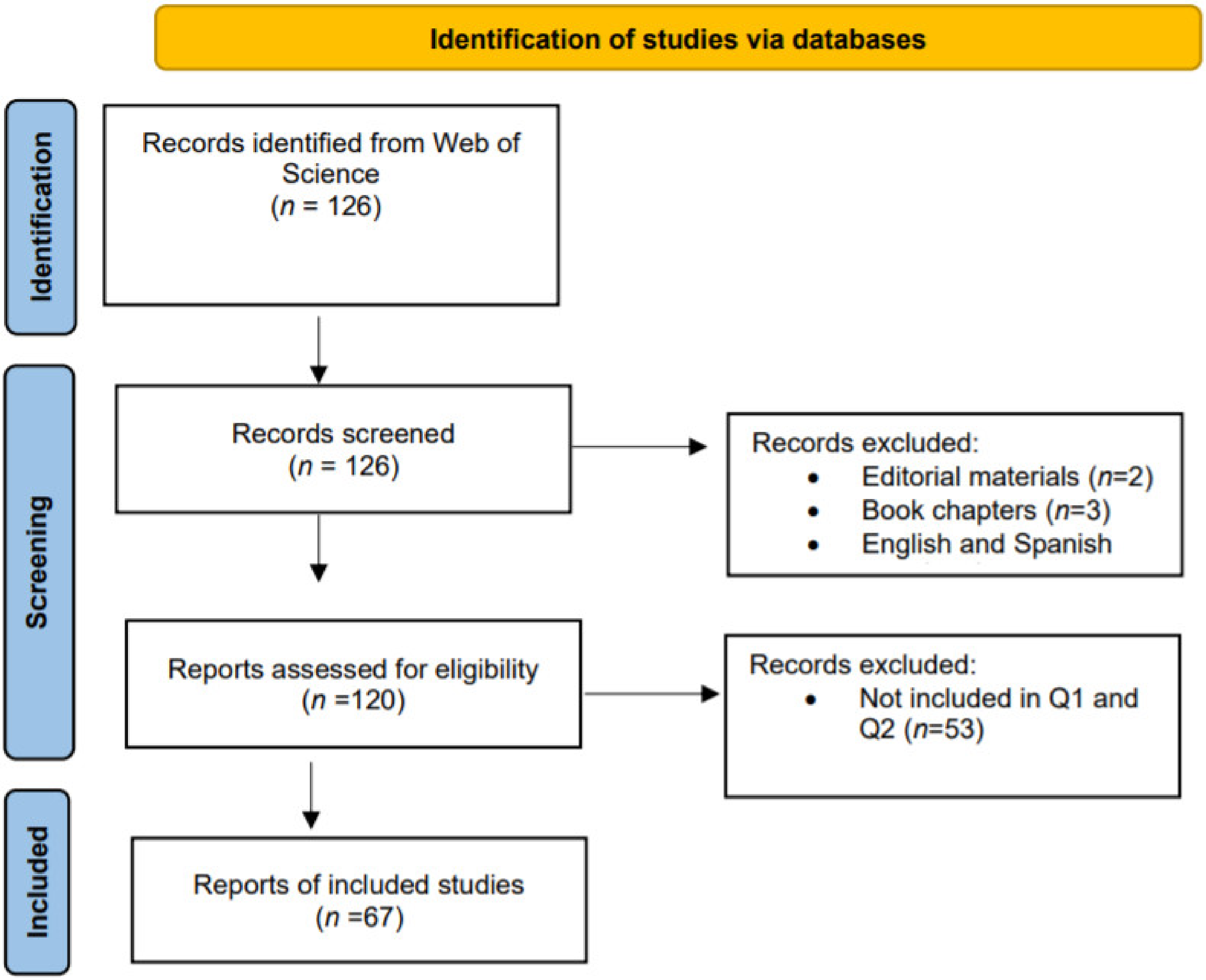

Figure S1. chart PRISMA.
